# Phytochemical analysis, in vitro and in silico effects from Alstonia boonei De Wild stem bark on selected digestive enzymes and adipogenesis in 3T3-L1 preadipocytes

**DOI:** 10.1186/s12906-023-04202-6

**Published:** 2023-10-20

**Authors:** Gabriel O. Anyanwu, Uju D. Ejike, Gideon A. Gyebi, Khalid Rauf, Nisar-Ur-Rehman, Jamshed Iqbal, Sumera Zaib, Usunomena Usunobun, Eusebius C. Onyeneke, Badriyah S. Alotaibi, Gaber El-Saber Batiha

**Affiliations:** 1 Department of Biochemistry, Bingham University, Karu, Nasarawa State Nigeria; 2 Department of Pharmacy, COMSATS University Islamabad, Abbottabad Campus, Pakistan; 3 Centre for Advanced Drug Research, COMSATS University Islamabad, Abbottabad Campus, Pakistan; 4 Department of Basic and Applied Chemistry, Faculty of Science and Technology, University of Central Punjab, Lahore, 54590 Pakistan; 5 Department of Biochemistry, Faculty of Basic Medical Sciences, Edo University Uzairue, Auchi, Edo State Nigeria; 6 Department of Biochemistry, University of Benin, Benin City, Edo State Nigeria; 7 Department of Pharmaceutical Sciences, College of Pharmacy, Princess Nourah Bint Abdulrahman University, Riyadh, 11671 Saudi Arabia; 8 Department of Pharmacology and Therapeutics, Faculty of Veterinary Medicine, Damanhour University, Damanhour, AlBeheira, 22511 Egypt

**Keywords:** Obesity, *Alstonia boonei*, Alkaloid, Saponin, Lipase, Amylase

## Abstract

**Background:**

Obesity is a global health issue arising from the unhealthy accumulation of fat. Medicinal plants such as *Alstonia boonei* stem bark has been reported to possess body weight reducing effect in obese rats. Thus, this study sought to investigate the in vitro and in silico effects of fractions from *Alstonia boonei* stem bark on selected obesity-related digestive enzymes and adipogenesis in 3T3-L1 preadipocytes.

**Method:**

Two fractions were prepared from *A. boonei*: crude alkaloid fraction (CAF) and crude saponin fraction (CSF), and their phytochemical compounds were profiled using Liquid chromatography with tandem mass spectrometry (LCMS/MS). The fractions were assayed for inhibitory activity against lipase, α-amylase and α-glucosidase, likewise their antiadipogenic effect in 3T3-L1 adipocytes. The binding properties with the 3 enzymes were also assessed using in silico tools.

**Results:**

Eleven alkaloids and six saponin phytochemical compounds were identified in the CAF and CSF using LCMS/MS. The CAF and CSF revealed good inhibitory activity against pancreatic lipase enzyme, but weak and good activity against amylase respectively while only CSF had inhibitory activity against α-glucosidase. Both fractions showed antiadipogenic effect in the clearance of adipocytes and reduction of lipid content in 3T3-L1 adipocytes. The LCMS/MS identified compounds (41) from both fractions demonstrated good binding properties with the 3 enzymes, with at least the top ten compounds having higher binding energies than the reference inhibitors (acarbose and orlistat). The best two docked compounds to the three enzymes were firmly anchored in the substrate binding pockets of the enzymes. In a similar binding pattern as the reference acarbose, Estradiol-17-phenylpropionate (-11.0 kcal/mol) and 3α-O-trans-Feruloyl-2 α -hydroxy-12-ursen-28-oic acid (-10.0 kcal/mol) interacted with Asp197 a catalytic nucleophile of pancreatic amylase. Estradiol-17-phenylpropionate (-10.8 kcal/mol) and 10-Hydroxyyohimbine (-10.4 kcal/mol) interacted with the catalytic triad (Ser152-Asp176-His263) of pancreatic lipase while Estradiol-17-phenylpropionate (-10.1 kcal/mol) and 10-Hydroxyyohimbine (-9.9 kcal/mol) interacted with Asp616 and Asp518 the acid/base and nucleophilic residues of modelled α-glucosidase.

**Conclusion:**

The antiobesity effect of *A. boonei* was displayed by both the alkaloid and saponin fractions of the plant via inhibition of pancreatic lipase and adipogenesis.

**Supplementary Information:**

The online version contains supplementary material available at 10.1186/s12906-023-04202-6.

## Introduction

Obesity is a global epidemic affecting both developed and developing countries [1]. Obesity is characterized by the uneven accumulation and distribution of fat usually accompanied by systemic inflammation [2]. Besides social stigmatization and health costs of obesity on individuals [3], obesity is a risk health factor for several disorders, such as hyperglycemia, type 2 diabetes mellitus, dyslipidemia, hypertension, osteoarthritis, heart attack, a few forms of cancer and mortality [4, 5].

Globally, in 2016, over 650 million adults aged 18 and above were obese representing about 13% of the adult world population but as for children and adolescents who were 5–19 years, over 340 million were overweight or obese in the same period [6]. According to the National Center for Health Statistics of the U.S. Centers for Disease Control and Prevention (CDC), the average American has put on 15 or more additional pounds without getting any taller since the late 1980s and early 1990s [7]. According to Hales et al. [8], the obesity rate increased to over 42% among adults in the USA from 2000 to 2018. Recent national data on obesity trends in Africa are scarce because fighting undernutrition and food insecurity has been the focus, although overnutrition and undernutrition co-exist in many African countries [9].

The path of balancing energy intake with adequate energy expenditure is highly recommended in the prevention of obesity. However, there are several options in the treatment or management of obesity, of which more than one way is recommended, these include physical activity (exercise), dieting, weight-loss medications, surgery, browning of white fat cells and hormonal treatment. According to Busetto et al*.* [10], most obesity management guidelines recommend anti-obesity medications and bariatric surgery in patients with higher body mass index (BMI) values (BMI ≥ 40 kg/m^2^) except in cases where obesity-related comorbidities are pertinent.

Current antiobesity medications vary in their mechanisms of action which include inhibition of digestive enzymes of fat or sugar from food, increase energy expenditure, appetite suppression, inhibition of adipogenesis/lipogenesis and stimulation of lipolysis/apoptosis. The development of antiobesity agents has been characterized by failures at preclinical or clinical phases, medical prescription restrictions, and outright bans or withdrawals due to adverse effects. Current antiobesity drugs approved by Food and Drug Administration (FDA) include orlistat, liraglutide, diethylpropion, phentermine, phendimetrazine, and benzphetamine, lorcaserin, bupropion/naltrexone and topiramate/phentermine [11, 12]. While lorcaserin has been disapproved in the USA in 2020 due to adverse effects, semaglutide was approved in 2021 as a glucagon-like peptide 1 receptor [12]. Many of these drugs are unavailable and/or rarely available in developing countries and where they could be found they are very expensive for lower- and middle-class citizens.

The search for less expensive, less side effects, very effective and available antiobesity drugs from medicinal plants is ongoing. In this study, the plant of interest *Alstonia boonei* De Wild is a specie of *Alstonia* which consists of over 40 species, native to tropical and subtropical Africa, Australia, Southeast Asia and Central America (www.theplantlist.org; https://powo.science.kew.org/results?q=Alstonia%20boonei). *Alstonia boonei*, commonly called *Alstonia* or cheese wood, is called égbū *or egbun* (Igbo), ahùn (Yoruba), Ukhu (Edo) and Ukpukunu (Urhobo) in Nigerian languages. It is used in African traditional medicine for the management and treatment of malaria, rheumatic pain, toothache, ulcer and inflammatory disorders [13]. Previous research has reported that *Alstonia boonei* possesses hypoglycemic activity [14], hypotensive activity [13] and antiobesity activity [15]. The ethanol extract of *A. boonei* stem bark has been reported to decrease body weight and fat mass as well as triglyceride levels in obese male Wistar rats [15]. However, the mode of action of the plant’s antiobesity effect as well as possible fractions/compounds within the plant with antiobesity properties have not been reported, thus this became the basis for the current study. This study investigates the in vitro and in silico effects of fractions from *Alstonia boonei* stem bark on selected obesity-related digestive enzymes and adipogenesis in 3T3-L1 preadipocytes.

## Materials and methods

### Chemicals

Orlistat (PHR1445-1G; Lot#LRAA2822) and Acarbose (PHR1253-500MG; Lot#LRAA9057) were purchased from Sigma-Aldrich Co., USA, and were used as reference drugs. Alpha amylase (A3176-1MU; Lot# SLCL3712) from porcine pancreas type VI-B, alpha glucosidase type I from bakers yeast (G0660-750UN), and lipase from porcine pancreas (L0382-100KU; Lot# SLCB 1687) were purchased from Sigma-Aldrich, St. Lious, MO USA. Also, p-Nitrophenyl butyrate (N9876-1G; Lot# SLBW9021) and morpholinepropanesulphonic acid MOPS (M1254-25G; Lot# BCBZ6901) were purchased from Sigma-Aldrich, St. Lious, MO USA.

### Identification and preparation of plant material

The fresh stem bark of *A. boonei* de Wild was garnered from an area in Umuekwune autonomous community, Imo State, Nigeria. Authentication of the plant and allocation of voucher number (GA134-7421) was done by Dr. Jerome O. Ihuma from Bingham University (Department of Biological Sciences), Nasarawa State, Nigeria. The experiments and field studies conducted on *A. boonei*, including the collection of plant material, were in adherence to relevant institutional, national, and international guidelines and legislations. The *A. boonei* stem bark was cut into tiny pieces, air-dried at 23–25 oC and crushed into powder. Exactly 6 kg of plant powder was macerated using 15L methanol for three days at room temperature and repeated twice. Filtration was done using Whatman No. 1 filter paper and the filtrate collected was evaporated to slurry using a rotary evaporator under reduced pressure at 40 °C. The methanol extract that yielded 1.29 kg (21.5%) was extracted with n-hexane to remove the oils resulting in defatted methanol extract.

### Preparation of saponin and alkaloid fractions

The crude alkaloid fraction (CAF) and crude saponin fraction (CSF) of *A. boonei* were obtained by the procedures reported by Sarker et al*.* [16]. Extraction of 500 g of defatted methanol extract was done with 1N H_2_SO_4_, and the solution was stirred till homogeneity is attained, then ammonia was added to achieve 7.0 pH in an ice-block container; thereafter partitioned with ethyl acetate (3 × 500 ml) using a separating funnel and the ethyl acetate partition was concentrated via evaporation at 50 °C using rotary evaporator to yield the 146 g (29.2%) crude alkaloid fraction (CAF). For saponins, 500 g of defatted methanol extract was dissolved in 500 ml distilled water and partitioned with n-butanol (3 × 500 ml). The aqueous partition was separated from the butanol partition using separating funnel. Thereafter, 93 g (18.6%) of CSF was precipitated by the addition of diethyl ether to the n-butanol partition and then collected after decantation and centrifugation.

### LC–MS/MS analysis

The chromatographic separations of diluted fractions were executed on Agilent 1290 Infinity LC system attached to Agilent 6520 Accurate-Mass Q-TOF mass spectrometer on ESI positive and negative modes. The LC–ESI–MS parameters and experiment were performed as previously described by Anyanwu et al. [17]. The CSF and CAF fractions with the concentration of 5 mg/ml were analysed using Agilent 1290 Infinity LC system coupled to Agilent 6520 Accurate-Mass Q-TOF mass spectrometer with dual ESI source for positive and negative polarity. The liquid column was Agilent eclipse XDB-C18 narrow-bore, 150 mm × 2.1 mm and 3.5-micron. Other LC parameters were set at 25°C column temperature, 4°C autosampler temperature and 0.5 mL/mins flowrate. The solvents used were 0.1% formic acid in water (A) and acetonitrile (B) with an injection volume of 1.0μL. The run and post run time were 25 and 5 min respectively. The MS parameters were as follows: source voltage was 3500 V, the fragmentor voltage and skimmer were 125 V and 65 V respectively and OCT 1 RF Vpp was 750 V. The drying gas was set at 10 L/min and the gas temperature at 300 °C. Analyses were carried out on full scan mode, 100 – 3200 mass range (m/z), and processing of acquired data was done with Agilent MassHunter Qualitative Analysis B.05.00.

### Determination of in vitro pancreatic lipase inhibitory activity

Pancreatic lipase inhibitory activity of the plant fractions was assayed using the protocol described by Kim et al*.* [18] with some modifications. The enzyme buffer was made by adding 1 mM EDTA pH 6.8 and 10 mM morpholinepropanesulphonic acid, while the assay buffer was Tris buffer. To each well was added 164μL of assay buffer, 6μL pancreatic lipase solution (1 mg of enzyme/mL of enzyme buffer), 20μL of either the plant fractions/orlistat at different concentrations (0, 2.35, 4.69, 9.38, 18.75, 37.5, 75, 150, and 300 μg/mL) and incubated for 10 min at 37 °C. Then, 10μL of 10 mM p-nitrophenylbutyrate in assay buffer was included and incubated at 37 °C for exactly 15 min. All tests were performed in triplicates and the absorbance was read at 405 nm using a microplate reader. Lipase inhibitory activity by the plant fractions was calculated from the formula below:

$$\mathrm{Lipase}\;\mathrm{inhibition}\;\left(\%\right)=100-\left(\left(\mathrm B-\mathrm b/\mathrm A-\mathrm a\right)\;\mathrm x\;100\right)$$

Where B = activity with inhibitor, A = activity without inhibitor, b = negative control with inhibitor and a = negative control without inhibitor.

### Determination of in vitro alpha amylase inhibitory activity

The starch-iodine test was used to assay for α-amylase inhibitory activity of plant fractions in a microtitre plate as reported by Xiao et al*.* [19]. Exactly 25μL of assay buffer, 0.02 M sodium phosphate buffer, 20μL of soluble starch solution (1%, w/v) and 20μL of plant extracts/acarbose (0, 7.81, 15.63, 31.25, 62.5, 125, 250, 500, 1000 µg/mL) were incubated at 37 °C for 5 min. Afterward, 15μL of amylase solution (6 mg/mL) was placed into each well and incubated at 37 °C for exactly 15 min. Thereafter, 20μL of 1 M HCl was included to stop the enzymatic reaction, and 100μL of iodine reagent (5 mM I_2_ and 5 mM KI) was added. The change in colour was noted and subsequently, absorbance was measured at 620 nm on a microplate reader. The calculation of percentage inhibition of alpha amylase:

$$\%\;\mathrm I\mathrm n\mathrm h\mathrm i\mathrm b\mathrm i\mathrm t\mathrm i\mathrm o\mathrm n=\left(\mathrm{Abs}\;\mathrm{of}\;\mathrm{test}/\mathrm{Abs}\;\mathrm{of}\;\mathrm{control}\right)\;\mathrm x\;100$$

### Determination of in vitro alpha-glucosidase inhibitory activity

The inhibition of alpha-glucosidase was determined by the protocol described by Johnson et al*.* [20]. Exactly 50µL of plant fractions/acarbose (0, 2.35, 4.69, 9.38, 18.75, 37.5, 75, 150, and 300 μg/mL), 50µL positive control, or 50µL reagent blank were added to a 96-well plate. Then, 100µL of a 1.0U/mL α-glucosidase buffer solution was included and incubated for 10 min at 25 °C. Then, 50µL of a 5 mM p-nitrophenyl-α-D-glucopyranoside solution was injected into each well and incubated for 5 min at 25 °C. Subsequently, absorbance was recorded at 405 nm before and after incubation. The percent inhibition was calculated relative to acarbose (positive control) and buffer solution only (negative control).

$$\%\mathrm I\mathrm n\mathrm h\mathrm i\mathrm b\mathrm i\mathrm t\mathrm i\mathrm o\mathrm n=\left[\left(\mathrm\Delta\mathrm a\mathrm b\mathrm s\mathrm C\mathrm o\mathrm n\mathrm t\mathrm r\mathrm o\mathrm l-\mathrm\Delta\mathrm a\mathrm b\mathrm s\mathrm E\mathrm x\mathrm t\mathrm r\mathrm a\mathrm c\mathrm t\right)/\mathrm\Delta\mathrm a\mathrm b\mathrm s\mathrm C\mathrm o\mathrm n\mathrm t\mathrm r\mathrm o\mathrm l\right]\;\mathrm x\;100$$

### Antiadipogenic properties of the plants on 3T3-L1 adipocytes

#### Cell culture and differentiation

3T3-L1 preadipocytes were obtained from ZenBio (Research triangle park, NC, USA) and experiments were performed using the manufacturer’s manual. Approximately, 5,000 cells/cm^2^ were placed in tissue culture treated culture ware using 3T3-L1 preadipocyte culture Medium (DMEM, high glucose, bovine calf serum (BCS), HEPES pH 7.4, penicillin, amphotericin B and streptomycin). The cells were maintained until they were 100% confluent in a 5% CO_2_ incubator at 37 °C for 7 days. The confluent cells were incubated for an additional 48 h, then differentiation was initiated by removal of the culture medium which was replaced with an appropriate volume of 3T3-L1 differentiation medium (DMEM/Ham’s F-12 medium (1:1, v/v), fetal bovine serum (FBS), HEPES pH 7.4, biotin, human insulin, pantothenate, dexamethasone, streptomycin, amphotericin B, penicillin, 3-Isobutyl-1-methylxanthine (IBMX) and PPARγ agonist). After incubation for 3 days, the differentiation medium was replaced with 3T3-L1 adipocyte maintenance medium (differentiation medium without IBMX and PPARγ agonist) and incubated for 7 days. Each medium was changed every other day for better performance.

#### Cell viability assay

Cell viability was assessed colorimetrically by an MTT (3-(4,5-dimetyl-2-thiazolyl)-2,5-diphenyltetrazoliumbromide) assay as described by Yang et al*.* [21]. Cells cultured in DMEM medium were treated with plant fractions (0 (control), 100 and 300 µg/mL) for 2 days. Thereafter, they were incubated with a 5 mg/mL MTT solution for 3 h. Then the cells were dissolved in 0.04 N HCl (in isopropanol) and the level of formazane formed was analyzed by reading absorbance at 570 nm against absorbance at 630 nm.

#### Oil Red O staining of intracellular triglycerides

Differentiated 3T3-L1 adipocytes were gently washed two times with phosphate buffered saline (PBS), fixed with formalin (10%) and incubated for 30 min. Formalin was removed and cells were washed twice with distilled water. The cells were stained with freshly diluted Oil Red O solution (0.5% Oil Red O in isopropanol) at room temperature for 1 h. Then the dye retained in the cells was eluted with isopropanol and quantified by reading the absorbance at 500 nm [22].

#### Lipolysis determination

Lipolysis was determined through the measurement of glycerol released into the medium following the manufacturer’s protocol (free glycerol reagent, ZenBio, Research triangle park, NC, USA). Briefly, 100 μg/mL of plant fractions were placed in plates containing differentiated 3T3-L1 adipocytes and incubated for 24 h. Then, 100 μL of the conditioned medium was incubated with 100 μL of free glycerol reagent for 15 min at 25 °C. Then glycerol released was quantified by reading absorbance of each well at 540 nm.

### Molecular docking studies of LCMS/MS identified compounds against α-amylase, lipase and α-glucosidase

#### Protein structure preparation

The retrieval of protein structures was from the Protein Data Bank (http://www.rcsb.org) for the deposited three-dimensional structures of *Porcine* pancreatic α-amylase (PPA) (PDBID: 1OSE), *Porcine* pancreatic lipase (PPL) (PDBID: 1LPB). The existing ligands and water molecules were removed from all the crystal structures while missing hydrogen atoms were added using MGL-AutoDockTools (ADT, v1.5.6) [23].

The 3D structure of s*accharomyces cerevisiae* α-glucosidase is not yet available; therefore, a homology model was used to predict the 3D structure. The protein sequence of S*accharomyces cerevisiae* α-glucosidase with accession no. GAX68588. was downloaded from the National Centre for Biotechnology Information (NCBI) database in a FASTA format. The FASTA sequence of the enzyme was subsequently used for the modeling. Briefly, a SWISS-MODEL template library (SMTL) search coupled with sequence alignment was implemented on Swiss model webserver (https://swissmodel.expasy.org/). A total of 1324 templates was identified. The list was filtered down to 50 and then 5 templates. Finally, s*accharomyces cerevisiae* a maltose bound oligo-1,6-glucosidase with PDB ID: 3A4A which shares 72.24% identical sequence with s*accharomyces cerevisiae* α-glucosidase [24] was selected as a template upon which the enzyme was contracted. The selected template for models had Global Model Quality Estimate (GMQE) scores of 0.95, 0.690 and 0.628, sequence coverage of 0.993, sequence identities of 72.41%, and sequence similarities of 0.544. The sequence alignment is presented in Figure SM2a. The model was refined by GalaxyRefine [25], a web-tool available on GalaxyWEB server (http://galaxy.seoklab.org/). The quality of the refined model was analyzed on ProSA [26] and ERRAT [27], while Ramachandran plots were generated on PROCHECK [28]. From the ERRAT plot analysis, the overall quality factor (OQF) of the model was 96.80% above the generally accepted score of > 50%, while the Ramachandran phi/psi torsion angles analysis shows that 99.3% of amino acid residues were in a favoured region with additional 11.1% in the allowed region, while 0.2% were in the disallowed region.

### Ligand preparation

The retrieval of Structure Data Format (SDF) of acarbose and orlistat (reference inhibitors), and 41 compounds identified by LCMS/MS analyses of the CAF and CSF of *A. boonei* from the PubChem database (www.pubchem.ncbi.nlm.nih.gov) preceded their conversion to mol2 chemical format employing Open babel [29]. Also, the compounds not found on the database were converted to mol2 chemical format after which they were drawn using ChemDraw version 19. Non-polar hydrogen molecules were merged with the carbons, while the polar hydrogen charges of the Gasteiger-type were assigned to atoms. Furthermore, ligand molecules were converted to dockable PDBQT format with the help of AutoDock Tools.

### Molecular docking of phytochemicals with a targeted active site

The method used in our previous studies was adopted for this study [30, 31]. The active site targeted molecular docking with the reference inhibitors and the LCMS/MS identified compounds against PPA, PPL and modeled α-glucosidase (mG) was performed using AutoDock Vina in PyRx 0.8 [32]. For the docking analysis, the ligands were imported and energy minimization was accomplished using OpenBabel [29] incorporated into PyRx 0.8. The energy minimization parameter and conjugate gradient descent used were the Universal Force Field (UFF) and optimization algorithm respectively. Although other parameters not mentioned were set at default, the binding site coordinates of the respective target enzymes PPA (Å), PPL (Å) and mG (Å) for the dimensions are: center_x (8.55, 22.62, 21.80), center_y (22.62, 27.66, -5.51), center_z (52.15, 51.07, 18.38), Size x (19.57, 25.00, 25.0), Size y (19.61, 20.29, 26.35) and Size z (17.29, 22.86, 25.0). Discovery Studio Visualizer version 16 was used to view the molecular interactions.

### Statistical analysis

The various experimental data were presented as the mean ± S.E.M and were analysed by One-Way ANOVA using SPSS version 20 and GraphPad Prism 5, then tested by Tukey’s Multiple Range Test to ascertain the statistical significance of difference amongst groups in various parameters. *P* < 0.05 was taken as the significant level.

## Results

### Compounds result of the LCMS/MS analysis

**Fig. 1:**
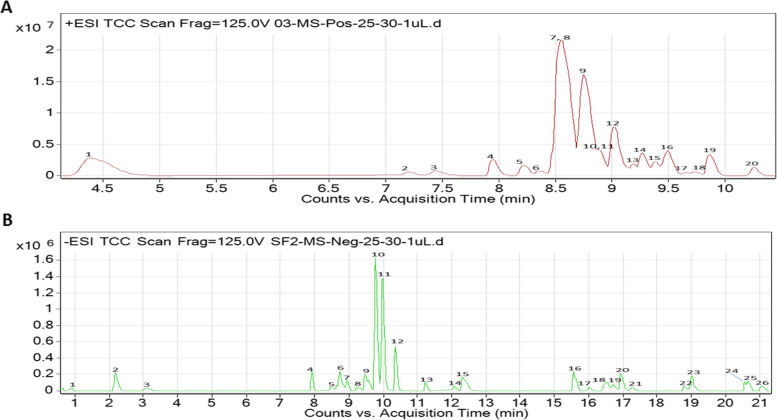
Total Compound Chromatogram of fraction of *A. boonei* via LCMS/MS analysis. **A** TCC of CAF of *A. boonei* via LCMS/MS analysis in positive polarity; **B** TCC of CSF of *A. boonei* via LCMS/MS analysis in negative polarity. The details of the numbers on each peak corresponding to the identified compounds from *A. boonei* are presented in Tables 1 and 2

Eleven alkaloids were identified in the CAF using LCMS (positive mode) and they include 10-Hydroxyyohimbine, 3-Hydroxyquinidine, 18-Hydroxyyohimbine, Ajmalicine, 16-Methoxy-2,3-dihydro-3-hydroxytabersonine, Vincamine, Aspidofractine, 16-Methoxytabersonine, Mitraphylline, Horhammericine and Catharanthine (Table 1; Fig. 1a, Table SM1a). Among the prominent peaks, three alkaloids were identified from the negative mode of the LCMS/MS analysis of CAF of *A. boonei*, and they are Cinegalline, Reserpic acid and Horhammericine (Figure SM1, Table SM1b; Table SM1c). Six saponin compounds majorly of the lactone and terpene classes were identified from the CSF of *A. boonei* using LCMS (negative mode) and they are Gardenoside, alpha-Carboxy-delta-decalactone, 5-Nonyltetrahydro-2-oxo-3-furancarboxylic acid, 3-O-cis-Coumaroylmaslinic acid, 3alpha-O-trans-Feruloyl-2alpha-hydroxy-12-ursen-28-oic acid and Lucidumol A (Table 2; Fig. 1b, Table SM2).

**Table 1:** Compounds result of the LCMS/MS analysis (positive mode) of Crude alkaloid fraction of *A. boonei*

Peak	RT	Mass	Name	Formula	Sub-class	Class
1	4.41	170.09	Furfural diethyl acetal	C_9_ H_14_ O_3_	Furans	Heterocyclic Compounds
2	7.21	370.19	10-Hydroxyyohimbine	C_21_ H_26_ N_2_ O_4_	Secologanin Tryptamine Alkaloids	Alkaloids
3	7.45	228.10	Depdecin	C_11_ H_16_ O_5_	Epoxide	Organic heterocyclic compound
4	7.95	358.13	Sweroside	C_16_ H_22_ O_9_	Iridoid Glycosides	Monoterpenes
5	8.22	340.18	3-Hydroxyquinidine	C_20_ H_24_ N_2_ O_3_	Cinchona Alkaloids	Alkaloids
6	8.47	370.19	18-Hydroxyyohimbine	C_21_ H_26_ N_2_ O_4_	-	Alkaloids
7	8.56	352.18	Ajmalicine	C_21_ H_24_ N_2_ O_3_	Monoterpenoid indole alkaloid	Alkaloids
8	8.56	384.21	16-Methoxy-2,3-dihydro-3-hydroxytabersonine	C_22_ H_28_ N_2_ O_4_	Monoterpenoid indole alkaloid	Alkaloids
9	8.63	354.19	Vincamine	C_21_ H_26_ N_2_ O_3_	Secologanin Tryptamine Alkaloids	Alkaloids
10	8.74	366.19	Aspidofractine	C_22_ H_26_ N_2_ O_3_	Indole alkaloid	Alkaloids
11	8.87	366.20	16-Methoxytabersonine	C_22_ H_26_ N_2_ O_3_	Indole alkaloid	Alkaloids
12	9.03	368.18	Mitraphylline	C_21_ H_24_ N_2_ O_4_	Indole alkaloid	Alkaloids
13	9.20	384.20	16-Methoxy-2,3-dihydro-3-hydroxytabersonine	C_22_ H_28_ N_2_ O_4_	Monoterpenoid indole alkaloid	Alkaloids
14	9.39	354.19	Vincamine	C_21_ H_26_ N_2_ O_3_	Secologanin Tryptamine Alkaloids	Alkaloids
15	9.47	368.17	Horhammericine	C_21_ H_24_ N_2_ O_4_	Indole alkaloid	Alkaloids
16	9.51	336.19	Catharanthine	C_21_ H_24_ N_2_ O_2_	Secologanin Tryptamine Alkaloids	Alkaloids
17	9.66	336.18	Catharanthine	C_21_ H_24_ N_2_ O_2_	Secologanin Tryptamine Alkaloids	Alkaloids
18	9.74	338.17	18-carboxy dinor Leukotriene B4	C_18_ H_26_ O_6_	Arachidonic Acids	Fatty acids and conjugates
19	9.87	404.24	Estradiol-17-phenylpropionate	C_27_ H_32_ O_3_	Steroids	-
20	10.26	366.19	Aspidofractine	C_22_ H_26_ N_2_ O_3_	Idole alkaloid	Alkaloids

**Table 2:** Compounds results of the LCMS/MS analysis (negative) of crude saponin fraction of *A. boonei*

Peak	RT	Mass	Name	Formula	Sub-class	Class
1	0.89	126.00	2-Hydroxyethanesulfonate	C_2_ H_6_ O_4_ S	Organosulfonic acids	Organic sulfonic acids and derivatives
2	2.17	202.08	Diethyl Oxalpropionate	C_9_ H_14_ O_5_	beta-keto acids and derivatives	Keto acids and derivatives
3	3.09	186.09	cis-2-Carboxycyclohexyl-acetic acid	C_9_ H_14_ O_4_	Dicarboxylic acid	Carboxylic acid
4	7.91	404.13	Gardenoside	C_17_ H_24_ O_11_	Iridoids	Monoterpenes
5	8.48	430.21	Cinegalline	C_23_ H_30_ N_2_ O_6_	Quinolizidine alkaloid	Alkaloid
6	8.73	156.08		C_4_ H_8_ N_6_ O		
7	8.84	204.10	Diethyl (2R,3R)-2-methyl-3-hydroxysuccinate	C_9_ H_16_ O_5_	Carboxylic ester	Ester
8	9.22	210.05	Vanilpyruvic acid	C_10_ H_10_ O_5_	Phenylpyruvic acid derivatives	Carboxylic acid
9	9.49	186.09	cis-2-Carboxycyclohexyl-acetic acid	C_9_ H_14_ O_4_	Dicarboxylic acid	Carboxylic acid
10	9.78	184.08	Glu-P-2	C_10_ H_8_ N_4_		
11	9.98	142.10	3-Methyl-2Z-heptenoic acid	C_8_ H_14_ O_2_	Fatty acids and conjugates	Fatty Acyls
12	10.36	186.09	cis-2-Carboxycyclohexyl-acetic acid	C_9_ H_14_ O_4_	Dicarboxylic acid	Carboxylic acid
13	11.23	272.16		C_14_ H_24_ O_5_		
14	12.07	214.12	alpha-Carboxy-delta-decalactone	C_11_ H_18_ O_4_	Delta valerolactones	Lactones
15	12.32	214.12	alpha-Carboxy-delta-decalactone	C_11_ H_18_ O_4_	Delta valerolactones	Lactones
16	15.57	458.34	18-acetoxy-1α-hydroxyvitamin D3 / 18-acetoxy-1α-hydroxycholecalciferol	C_29_ H_46_ O_4_		
17	16.01	256.17	5-Nonyltetrahydro-2-oxo-3-furancarboxylic acid	C_14_ H_24_ O_4_	Gamma butyrolactones	Lactones
18	16.56	618.39	3-O-cis-Coumaroylmaslinic acid	C_39_ H_54_ O_6_	Triterpenoids	Terpenes
19	16.74	648.40	3α-O-trans-Feruloyl-2α-hydroxy-12-ursen-28-oic acid	C_40_ H_56_ O_7_	Triterpenoids	Terpenes
20	16.95	278.15		C_17_ H_18_ N_4_		
21	17.28	298.25		C_18_ H_34_ O_3_		
22	18.80	328.26	MG(0:0/16:1(9Z)/0:0)	C_19_ H_36_ O_4_		
23	19.01	456.36		C_27_ H_52_ O_3_ S		
24	20.56	356.17		C_19_ H_24_ N_4_ O S		
25	20.68	382.18	1,2,10-Trihydroxydihydro-trans-linalyl oxide 7-O-beta-D-glucopyranoside	C_16_ H_30_ O_10_	O-glycosyl compounds	Carbohydrates and carbohydrate conjugates
26	21.06	472.36	Lucidumol A	C_30_ H_48_ O_4_	Triterpenoids	Terpenes

### Effect of fractions on α-amylase, lipase and α-glucosidase activities

**Fig. 2:**
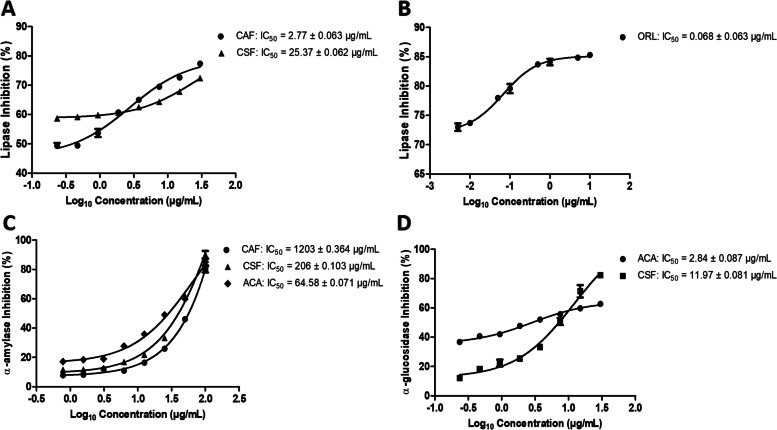
Inhibitory enzyme activities of *A. boonei* fractions. **A** & **B** are lipase activities, **C** is α-amylase and **D** is α-glucosidase activity. CAF – crude alkaloid fraction, CSF – crude saponin fraction, ORL- Orlistat and ACA—Acarbose. Values are mean ± SEM

The CSF fraction of *A. boonei* had moderate inhibitory activity against pancreatic lipase with an IC_50_ value of 25.37 ± 0.062 µg/mL, while CAF had better inhibitory activity against pancreatic lipase (IC_50_ = 2.77 ± 0.063 µg/mL), however, Orlistat (IC_50_ = 0.068 ± 0.063 µg/mL) displayed the highest inhibitory activity against pancreatic lipase (Table 3, Fig. 2A and B). The screening of CAF and CSF of *A. boonei* for anti-amylase activity showed weak and good inhibitory activity against alpha amylase respectively, but only the CSF of *A. boonei* showed potent α-glucosidase inhibitory activities (Table 3, Fig. 2C and D).

**Table 3:** Calculated IC50 values of fractions on α-amylase, lipase and α-glucosidase activities

*Alstonia boonei* fractions	α-amylase		Pancreatic lipase		α-glucosidase	
	Inhibition (%)	IC50 (μg/mL)	Inhibition (%)	IC50 (μg/mL)	Inhibition (%)	IC50 (μg/mL)
**ME**	40.37 ± 2.43	-	41.23 ± 1.85	-	32.56 ± 5.28	-
**CFF**	44.64 ± 4.32	-	37.98 ± 1.53	-	45.92 ± 5.06	-
**CAF**	74.54 ± 1.40	1203 ± 0.364	65.42 ± 2.19	2.77 ± 0.063	42.49 ± 1.63	-
**CSF**	74.97 ± 3.19	206 ± 0.103	60.21 ± 4.16	25.37 ± 0.062	77.95 ± 4.29	11.97 ± 0.081
**Orlistat**	NA	NA	93.18 ± 2.97	0.068 ± 0.063	NA	NA
**Acarbose**	83.18 ± 2.97	64.58 ± 0.071	NA	NA	76.70 ± 2.63	2.84 ± 0.087

*Alstonia boonei* fractions: *ME* methanol extract, *CAF* crude alkaloid fraction, *CSF* crude saponin fraction. Results of inhibition (%) are demonstrated as Mean ± SD of 3-replicates of every fraction. IC50 values are mean ± SEM; (–) fractions/extract did not inhibit 50% of enzyme activity at 1000 μg/mL; *NA* not applicable

### Antiadipogenic and lipolytic properties of the plants on 3T3-L1 preadipocytes

**Fig. 3:**
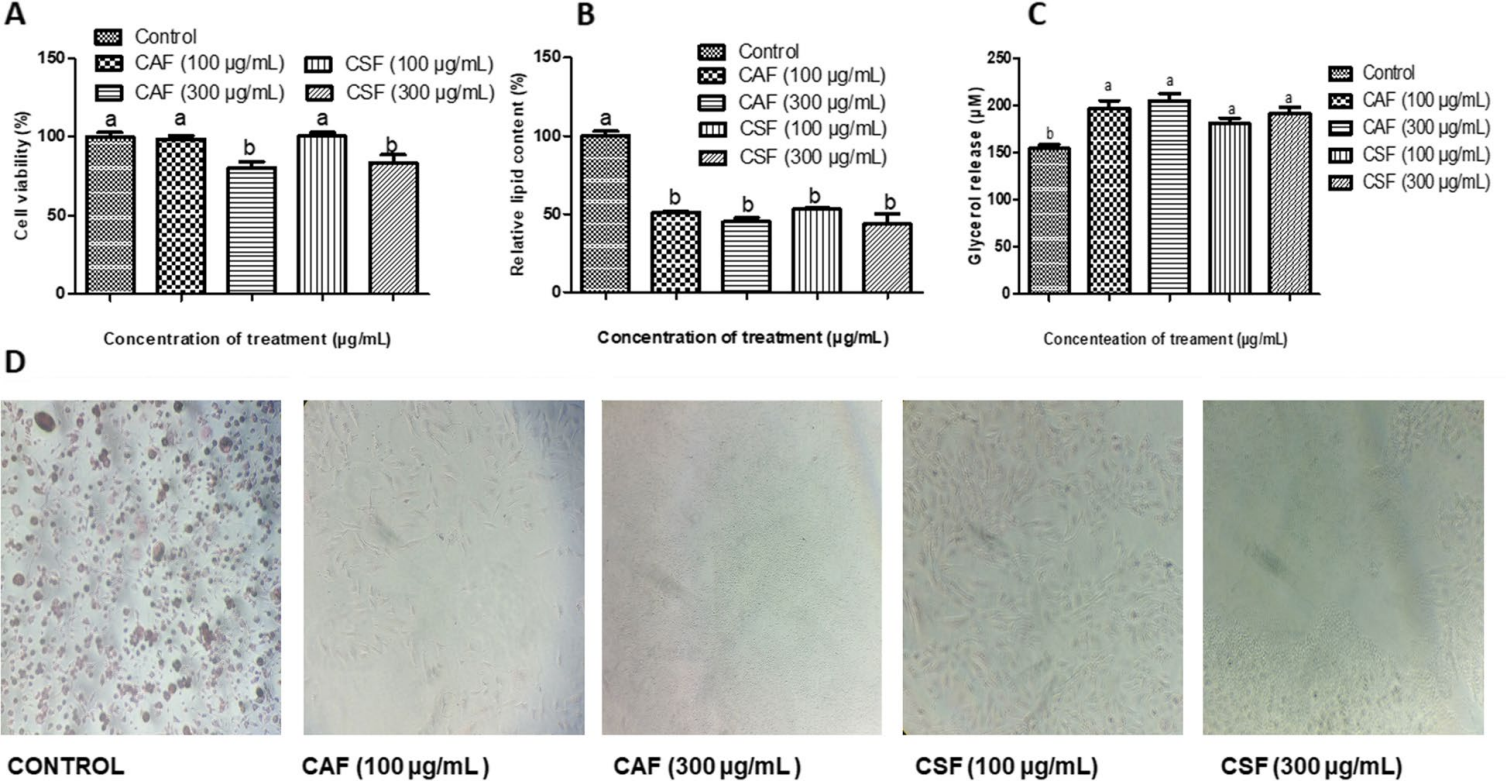
Effect of the fractions of *A. boonei* on differentiated 3T3-L1 adipocytes. **A** cell viability; **B** relative lipid content; **C** glycerol release; **D** adipocytes after staining with Oil Red O. Control is at 0 mg/dL of plant fractions; *n*=3

Apparently, no significant change (*p* > 0.05) in the cell viability of CAF and CSF of *A. boonei* at 100 µg/mL was observed when compared with the control, however, that of 300 µg/mL of CAF and CSF were significantly decreased with 80.18% and 83.15% viability respectively, and they were included in the study (Fig. 3a). The relative lipid content was expressed as the percentage of the control. In Fig. 3b, the lipid content of the cell treated with CAF and CSF decreased significantly (*p* < 0.05) in comparison to the control. However, the glycerol released by the cell as induced by CAF and CSF of *A. boonei* at 100 µg/mL was significantly elevated (*p* < 0.05) compared to the control (Fig. 3c). The visualized cells with Oil-Red O stain depicted lipid droplets as red areas within the cytoplasm (Fig. 3d). It was observed that the number and size of lipid droplets in the groups treated with CAF and CSF (100 and 300 µg/mL) were lower compared to the control. Also, the fraction-treated groups did not show a spherical shape of a matured adipocyte and the stain was rarely retained which might be signs of inhibition of differentiation.

### Molecular docking results

#### Molecular docking of identified compounds against human α-amylase, lipase and α-glucosidase

**Fig. 4:**
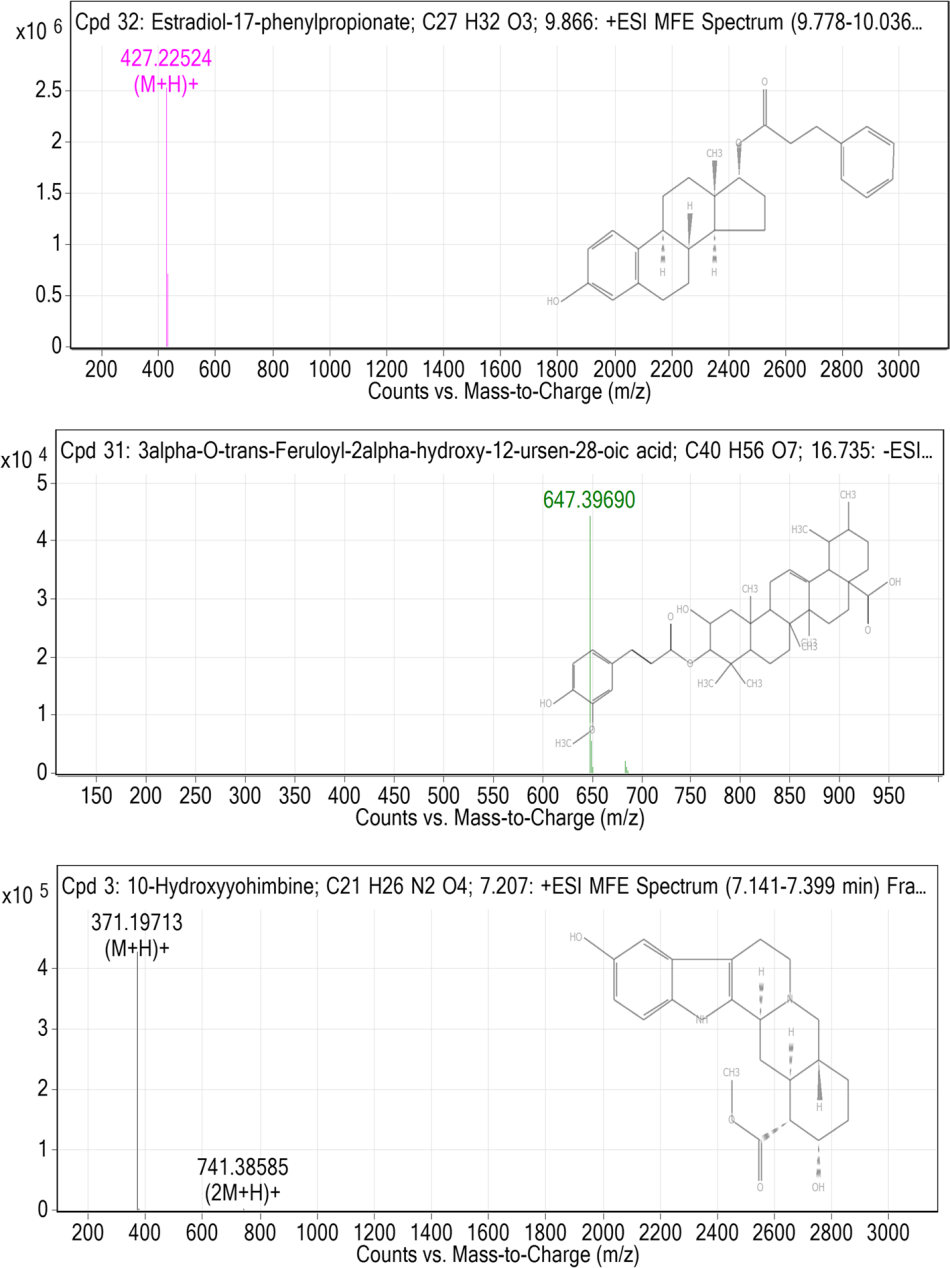
Top docked compounds of *A. boonei* fractions. Compounds from CAF are estradiol-17-phenylpropionate and 10-hydroxyyohimbine, and from CSF is 3α-O-trans-Feruloyl-2α -hydroxy-12-ursen-28-oic acid

The binding affinities from the docking analysis of the 41 compounds identified by the LCMS/MS analysis of the CAF and CSF of *A. boonei* and the reference compounds (acarbose and orlistat) against PPA, PPL and mG are shown in (Table SM3). Based on the minimum binding energies, binding poses, and interaction in the catalytic site, the top two ranked compounds for each enzyme were selected for interactive analysis. After ranking, it was observed that at least the top ten docked compounds to the three enzymes had binding energies higher than the reference inhibitors (Table SM3). For the validation of the docking protocol, the co-crystalized inhibitor (acarbose) was docked into the binding site of PPA, with a binding energy of -7.9 kcal/mol. Estradiol-17-phenylpropionate (-11.0 kcal/mol) and 3α-O-trans-Feruloyl-2α-hydroxy-12-ursen-28-oic acid (-10.0 kcal/mol) an identified constituent of the CAF and CSF respectively had the topmost binding energies to PPA (Fig. 4). Orlistat the reference inhibitor and the top two ranked compounds (Estradiol-17-phenylpropionate and 10-Hydroxyyohimbine) as in Fig. 4 were docked into the active site of PPL with the binding energies of -6.7, -10.8 and -10.2 kcal/mol respectively.

Estradiol-17-phenylpropionate (-1010 kcal/mol) and 3α-O-trans-Feruloyl-2 α -hydroxy-12-ursen-28-oic acid (-10.0 kcal/mol) were identified as the two top docked compounds to the binding pockets of the modelled scG with higher binding energies than the reference inhibitor (acarbose, -7.9 kcal/mol). Estradiol-17-phenylpropionate demonstrated the highest binding tendencies to the three enzymes (PPA, PPL and scG), thereby exhibiting multi-binding potential, while 3α-O-trans-Feruloyl-2alpha-hydroxy-12-ursen-28-oic_acid demonstrated high binding tendencies to PPA and scG.

#### Amino acid interaction of top docked compounds with α-amylase, lipase and α-glucosidase

**Fig. 5:**
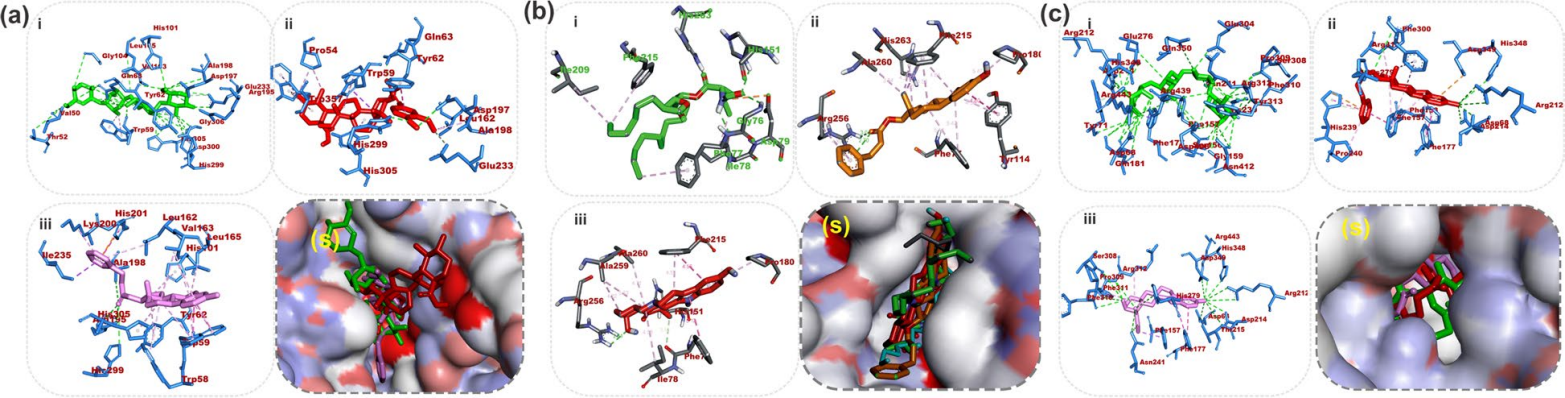
Details of binding mode: (s) solvent-accessible surface view (i-iii) Interaction view of ligands in binding pocket of human pancreatic alpha-amylase (a), human pancreatic lipase (b) and human lysosomal α-glucosidase (c). Colours indicate the stick representations of ligands for **(a)** as **(i)** green: acarbose (reference inhibitors)** (ii)** gold: 3-O-cis-coumaroylmaslinic_acid **(iii) **red: Furfural diethyl acetal. Colours indicate the stick representations of ligands for **(b)** as **(i)** green: orlistat (referencer inhibitors)** (ii)** gold: estradiol-17-phenylpropionate **(iii)** red: 10-hydroxyyohimbine **(iv)** cyan: methoxyundecyl phosphonic acid (MUP) (*interaction not shown)*. Colours indicate the stick representations of ligands for **(c)** as **(i)** green: acarbose (referencer inhibitors)** (ii)** gold: 3α-O-trans-feruloyl-2α-hydroxy-12-ursen-28-oic acid **(iii)** red: estradiol-17-phenylpropionate

The amino acid interaction of the combined list of the two lead compounds and reference inhibitors to the catalytic residues of PPA, PPL and scG is represented in Table 4. The interaction of respective ligand groups with residues of the enzymes was majorly through hydrophobic and few H-bonding below (less than 3.40 Å). A surface representation and 3D interaction of the lead compounds and reference inhibitors in the binding pockets of the enzymes have been represented in Fig. 5. The orientation of acarbose was stretched into the binding pocket of PPA. Estradiol-17-phenylpropionate and 3α-O-trans-Feruloyl-2α-hydroxy-12-ursen-28-oic acid were docked in close orientation as acarbose to the enzyme. The cyclopenta[a]phenanthren-17-yl moiety of estradiol-17-phenylpropionate was orientated towards the hydrophobic gate interacting with several residues (through alkyl and Pi-alkyl interaction. The same moiety formed Pi-Pi stacking with Trp59 of the hydrophobic gate. The phenyl ring of 3-phenylpropanoate moiety formed a Pi-cation and Pi-sigma contact with His201 and Ile235, while the proponoate functional group formed H-bond with His299, Arg195, and Ala198. The bulk ursen-28-oic acid moiety of 3α-O-trans-Feruloyl-2α-hydroxy-12-ursen-28-oic acid was directed orientated into the pocket towards the hydrophobic gate, also interacting with Trp59 through Pi-Sigma contact, while the feruloyl formed H-bonds with Gln63, Asp197 and His299 (Fig. 5aiii and Table 4).

**Table 4:** Interaction of amino acid residues of α-amylase, lipase and α-glucosidase with the top two LCMS/MS identified compounds from the crude alkaloid and saponin fraction of *A. boonei*

**Compounds**	**Binding** **Energies** **(Kcal/mol)**	Protein		Hydrogen bonds (Bond distance (Å))		Hydrophobic Interaction (Bond distance (Å))	Other interactions (Bond distance (Å))	
			**Numbers**	**Interacting residues**	**Numbers**	**Interacting residues**	**Numbers**	**Interacting residues**
Acarbose	-7.3	PPA	21	Thr 52 Val50 Trp59 Gln63(3) Tyr62 Leu165 His101 Thr163 Ala 198 His305(2) Asp197 Gly306(2) His299 Arg195 Glu233(2) Asp300	1	Trp59		
Estradiol-17-phenylpropionate	-11		3	His299 Arg195 Ala198	11	Tyr62 Ile235 Lys200 Leu162 Trp59 His395 Leu165 Val163 Trp58 Trp62 His101	2	His201 Ile235
3α-O-trans-Feruloyl-2 α -hydroxy-12-ursen-28-oic acid	-10.0		3	His299 Asp197 Gln63	6	Pro54 Trp357 Tyr62 Leu162 His305 Glu233	1	Trp59
Orlistat	-6.7	PPL	4	Phe77 Gly76 His151His263	3	Ile78 Ile209 Phe215	1	Asp79
Estradiol-17-phenylpropionate	-10.8		1	Cys256	10	Phe77(2) Tyr114(2) Pro180 Phe215(2) Ala260 His263(2)		none
10-Hydroxyyohimbine	-10.4		3	Phe77 His151Arg256	8	Phe77 Ile78 Pro180 Phe215(2) Ala260(2) Ala259		none
Acarbose	*-7.3*	mG	23	Asp68 Tyr71 Gln181 Asp214 Arg443 Arg439 His348 Glu276 Arg212 Gln350 Glu304 Arg312 His239 Asn241 Ser156 Tyr313 Phe157 Gly156 Asn412 Phe310 Pro309 Ser308 Asp408	1	Phe177		none
Estradiol-17-phenylpropionate	-10.1		6	Asp68 Arg212 His358 Asp214 His279 Arg312	5	Pro240 Phe157 Phe177 Phe158 Phe300	2	His239 Asp349
10-Hydroxyyohimbine	-9.9		13	Asp68 Thr215 Arg443 Arg212 His348 Asp214 Asp349 Phe310 Arg312 Pro309 His279 Ser308 Asn241	3	Phe157 Phe177 Phe311		none

The result of the validation of the docking protocol for the PPL enzymes using the co-crystalized inhibitor C11 alkyl phosphonate (methoxyundecyl phosphonic acid (MUP)) shows that all conformations were located in the active site. Thereafter, the best conformation formed was compared with interactions of the lowest binding energy pose of orlistat the reference inhibitor used in this study. Orlistat was docked into the same domain as MUP (Fig. 5bS). Orlistat displayed a strong binding to PPL with four hydrogen bonds and 3 hyrdophobic interactions. Importantly, the formamido-4-methylpentanoate of orlistat was mainly responsible for forming these four hydrogen bonds (Fig. 5bi). In the case of estradiol-17-phenylpropionate to top docked compounds to PPL the interactions were stabilized by a hydrogen bond between the carbonyl moiety of the phenylpropionate group and Arg256. While the estradiol rings are formed all the Pi-Pi stackings, Pi-Alkyl and akyl interactions with catalytic residues (Fig. 5bii). The 17-hydroxyl group of 10-Hydroxyyohimbine formed a hydrogen bond with Arg265, while the carbonyl oxygen and methoxy group of the carboxylate end formed Hydrogen bonds with His151 and Phe77 respectively (Fig. 5biii). The interaction was further stabilized by hydrophobic contacts with other residues listed in Table SM4.

Acarbose an inhibitor of the α-glycosidase was docked into the binding pocket as predicted by mG. Acarbose formed several H-bonds with the residues in the binding pocket of the enzymes with few hydrophobic interactions. Notable among such residues is Asp68. The two lead compounds interacted in a similar pattern with the same amino acid residues as acarbose. The hydroxyl group on the cyclopenta[a]phenanthren-17-yl moiety of estradiol-17-phenylpropionate formed 6 H-bonds with Asp68 Arg212 His358 Asp214, while the 2 other H-bonds with His279 and Arg312 was formed by the propanoate group that is linked to phenyl ring of 3-phenylpropanoate moiety (Fig. 5Cii). The 7 and 18 hydroxyl group attached to the dodecahydroyohimbane core or 10-hydroxyyohimbine and the carboxylate group was responsible for the several H-bonds between 10-hydroxyyohimbine and the residues of the binding pocket (Fig. 5Ciii). The interaction was stabilized by other Pi-Alkyl and Alkyl contacts between aglycon and the rest residues (Table 4).

## Discussion

Majority of the compounds identified from CAF of *A. boonei* were alkaloids and they are Cinegalline, Reserpic acid, Horhammericine, 10-Hydroxyyohimbine, 3-Hydroxyquinidine, 18-Hydroxyyohimbine, Ajmalicine, 16-Methoxy-2,3-dihydro-3-hydroxytabersonine, Vincamine, Aspidofractine, 16-Methoxytabersonine, Mitraphylline, and Catharanthine. Six saponin compounds majorly of the lactone and terpene classes were identified from the CSF of *A. boonei* and they are Gardenoside, alpha-Carboxy-delta-decalactone, 5-Nonyltetrahydro-2-oxo-3-furancarboxylic acid, 3-O-cis-Coumaroylmaslinic acid, 3alpha-O-trans-Feruloyl-2alpha-hydroxy-12-ursen-28-oic acid and Lucidumol A.

Reserpic acid has been shown to have activity against pancreatic lipase through molecular docking [33]. Yohimbine was reported to be effective in stimulating lipid mobilization in obese and non-obese subjects [34]. Also, yohimbine induced weight loss by acting as an α2-AR antagonist [35]. Sweroside has shown α-glucosidase inhibitory activities [36] while Vincamine displayed hypoglycaemic and hypolipidemic activity [37].

Pancreatic lipase is the major enzyme that facilitates the digestion and absorption of triglycerides in the gastrointestinal tract. Thus, inhibitors of pancreatic lipases are proposed to function as antiobesity agents [38]. The CAF and CSF fractions of *A. boonei* inhibited the activity of pancreatic lipase, although, this is the first time the lipase inhibitory activity for *A. boonei* is reported. The inhibition of pancreatic lipase by CAF and CSF of *A. boonei* possibly led to decreased body weight and white adipose tissue in the treated rats. The reduced absorption of ingested dietary fat leads to an overall reduced caloric absorption, thereby leading to weight loss.

Pancreatic lipase inhibitors from medicinal plants have gained considerable attention owing to their wide range of sources, structural diversity and possible low toxicity [38]. These natural products are of different classes of phytochemicals such as polysaccharides, polyphenols, terpene trilactones, flavonoids, alkaloids, saponins and carotenoids. For example, a flavonoid-rich extract of *Cassia fistula* and *Solanum nigrum* inhibited pancreatic lipase activity [39, 40]. The alkaloid rich fraction (CAF) and saponin rich fraction (CSF) of *A. boonei* inhibited pancreatic lipase, and this effect has been found among some alkaloids and saponins of other plants [41].

Targeting carbohydrate hydrolysing enzymes is an approach used to assess the efficacy of drugs to manage or treat obesity and some drugs for obesity function by inhibiting α-amylase. α-Amylase inhibitors have great potential to treat obesity [42]. The saponin fraction of *A. boonei* showed good inhibition of α-amylase although not better than Acarbose. This indicates that *A. boonei* might have its anti-amylase properties resident in CSF but CAF might not exert antiobesity activities by inhibiting α-amylase activity or that the active ingredients might have been masked by other compounds in the in vitro study. Only the saponin fraction from *A. boonei* showed strong α-glucosidase inhibitory activity suggesting that the plant may have potent blood glucose lowering properties when consumed which was observed in the in vivo studies as rats treated with CSF revealed reduced blood glucose levels.

The treatment of 3T3-L1 preadipocytes with 100 and 300 µg/mL of CAF and CSF of *A. boonei* produced viable cells, although, at 300 µg/mL of both fractions, values were lower than the control cells which indicated a slight decrease in the rate of cell metabolic activity. However, since the values were closer to the control, the 80% viability for cells produced by CAF and CSF at 300 µg/mL were considered viable [43], however, it is important to note that doses above 300 µg/mL might pose relatively low cytotoxicity to 3T3-L1 preadipocyte cells.

In assessing the lipid accumulation using the Oil Red O Staining, CAF and CSF of *A. boonei* inhibited adipocyte differentiation in the cells. The inhibition of adipogenesis caused by the fractions was reflected in the reduced relative lipid content of the cells compared to the control. This reflects a reduction in the quantity of triglycerides in the 3T3-L1 cells [44] The effect of *A. boonei* in preventing lipid accumulations shows its antiobesity potential. Lipolysis plays a central role in the regulation of energy balance because it is the process whereby triglycerides are hydrolyzed into glycerol and free fatty acids [45]. The increased glycerol released by the CAF and CSF of *A. boonei* indicates that they possess the ability to enhance lipid metabolism, particularly breaking down of triglycerides leading to glycerol release by the adipocytes. The facilitation of lipolysis is an important factor in the search for antiobesity agents.

The best mode of interaction between known ligands and protein(s) could be predicted by structure-based virtual screening using the scoring functions to determine the force of non-covalent interactions between a molecular target and ligand. This method has been employed in the search for natural inhibitors of PPA, PPL and mG [46]. The LCMS/MS identified compounds in the fractions demonstrated good binding properties with the 3 enzymes, with at least the top ten compounds having higher binding energies than the reference inhibitors. The biochemical studies of amylase and the X-ray crystallographic analysis have revealed that the catalytic triad includes Asp300, Glu233 and Asp197 [47, 48]. Asp300 has been reported to optimize the orientation of the substrate in the binding site, Asp197 mediates in the nucleophilic reaction [47], while residue Glu233 facilitates acid–base catalysis [30]. The two top docked compounds to PPA demonstrated a similar binding pattern as the reference inhibitor. Within the active of PPA Asp197 has been reported to be mainly responsible for the cleavage of the glycosidic bonds in polysaccharides. The critical function of Asp197 as a catalytic nucleophile in hydrolytic reactions for polymeric substrates such as edible starch and the interaction of this residue with known inhibitors has been reported [49, 50]. Although there is a paucity of information on the inhibitory role of furfural diethyl acetal and 3-O-cis-Coumaroylmaslinic acid on α-amylase activities, the aglycon unit (maslinic acid) of 3-O-cis-Coumaroylmaslinic acid has been widely documented as a natural antidiabetic compound which could reduce the levels of blood glucose [51, 52]. Several inhibitory mechanisms that underpin the mechanism of known alpha-amylase inhibitors have been documented such as the effectiveness to form H-bonds and other interactions with the residues of catalytic triad and other catalytic residues, interactions at other sites different from the active site, or the formation of covalent bond through an epoxy or aziridine group with the enzyme has been reported as one of the common inhibitory mechanisms [53, 54].

The glycoprotein PPL consists of the C region (residues from 336 to 449) and the N region (residues from 1 to 335), and in the latter, the active site consists of residues from 247 to258 with the catalytic triad (Ser152-Asp176-His263) [55]. Estradiol-17-phenylpropionate and 10-Hydroxyyohimbine the top two docked compounds to PPL interacted with the residues that make up the catalytic triad in a similar docked pose as orlistat and MUP indicating their structural similarity in the enzyme active site [56]. They also, interacted with Phe77, an important amino acid for lipase activity [56, 57], they, therefore stand out as a potential candidate for inhibiting the PPL catalytic activity. Also, the aglycon unit (estradiol and yohimbine) of estradiol-17-phenylpropionate and 10-Hydroxyyohimbine respectively have been reported to manifest interesting *anti*-diabetic and anti-obesity activities [34, 58].

The substrate-binding pocket of mG is located near the C-terminal ends of β-strands of the catalytic (β/α)8 domain [59]. As a notable member of the GH31 family enzymes that execute catalysis through a classical Koshland double displacement reaction mechanism with retention of the anomeric carbon configuration in the product [59]. 10-Hydroxyyohimbine and estradiol-17-phenylpropionate the top docked compounds to the mG enzyme exhibited a similar binding mode as the reference inhibitor (acarbose) which is a competitive inhibitor of mG. They interacted with important residues of the binding pocket as predicted in as identified in a similar study [60]. Overall, evidence from previously reported activities of the top docked compounds to the three enzymes and results from this in silico study suggest that the activities of the fractions may be as a result of the synergistic activities of, at least, the top two-ranked compounds identified from the molecular docking analysis.

## Conclusion

The CAF and CSF of *A. boonei* inhibited pancreatic lipase and adipogenesis, decreased lipid content and induced lipolysis in 3T3-L1 adipocytes. Among the compounds identified from fractions of *A. boonei*, estradiol-17-phenylpropionate and 3α-O-trans-Feruloyl-2α -hydroxy-12-ursen-28-oic acid are very good inhibitors of PPA, estradiol-17-phenylpropionate and 10-hydroxyyohimbine for PPL, while 10-Hydroxyyohimbine and estradiol-17-phenylpropionate for mG. Hence, the antiobesity activities of *A. boonei* may be as a result of the activities of, at least one or more of the compounds.

### Supplementary Information

**Additional file 1: Figure SM1.** Total Compound Chromatogram of crude alkaloid fraction of A. boonei via LCMS/MS analysis in negative polarity. The details of the numbers on each peak corresponding to the identified compounds from A. boonei are presented in Table SM1b. **Figure SM2a.** Saccharomyces cerevisiae α-glucosidase aligned with the template protein. **Figure SM2b.** Ramachandran phi/psi torsion angles plot of modelled Saccharomyces cerevisiae α-glucosidase. **Figure SM2c.** Errat plot of modeled Saccharomyces cerevisiae α-glucosidase.

**Additional file 2: Table SM1a.** Chromatographic analysis of crude alkaloid fraction of A. boonei via positive polarity. **Table SM1b.** Compounds result of the LCMS/MS analysis (negative mode) of crude alkaloid fraction of A. boonei. **Table SM1c.** Chromatographic analysis of crude alkaloid fraction of A. boonei via negative polarity. **Table SM2.** Chromatographic analysis of crude saponin fraction of A. boonei via negative polarity. **Table SM3.** Binding energies of LCMS/MS identified compounds from the fractions of A. boonei docked in the active sites of human α-amylase, lipase and α-glucosidase. **Table SM4.** Top two ranked compounds from the molecular docking of the LCMS/MS identified compounds from the crude.

## Data Availability

The data that support the findings of this study are available in supplementary file.

## Abbreviations

BCS: Bovine calf serum
BMI: Body mass index
CAF: Crude alkaloid fraction
CDC: U.S. Centers for Disease Control and Prevention
CSF: Crude saponin fraction
DMEM: Dulbecco's Modified Eagle Medium
FDA: Food and Drug Administration
GMQE: Global Model Quality Estimate
LCMS/MS: Liquid chromatography with tandem mass spectrometry
mG: Modeled α-glucosidase
MTT: 3-(4,5-Dimetyl-2-thiazolyl)-2,5-diphenyltetrazoliumbromide
OQF: Overall quality factor
PBS: Phosphate buffered saline
PPA: Porcine pancreatic α-amylase
PPARγ: Peroxisome proliferator-activated receptor gamma
PPL: Porcine pancreatic lipase
SDF: Structure Data Format
UFF: Universal Force Field

## References

[1] World Health Organization (2000). Report of the commission on ending childhood obesity.

[2] González-Muniesa P, Mártinez-González MA, Hu FB, Després JP, Matsuzawa Y, Loos RJF, Moreno LA, Bray GA, Martinez JA (2017). Obesity. Nat Rev Dis Primers.

[3] Sagi-Dain L, Echar M, Paska-Davis N (2022). Experiences of weight stigmatization in the Israeli healthcare system among overweight and obese individuals. Isr J Health Policy Res.

[4] Bosy-Westphal A, Müller MJ (2021). Diagnosis of obesity based on body composition-associated health risks-time for a change in paradigm. Obes Rev.

[5] Pedrosa MR, Franco DR, Gieremek HW, Vidal CM, Bronzeri F, de Cassia RA, de Carvalho Cara LG, Fogo SL, Eliaschewitz FG (2022). GLP-1 agonist to treat obesity and prevent cardiovascular disease: what have we achieved so far?. Curr Atheroscler Rep.

[6] World Health Organization. Report of the commission on ending childhood obesity. Geneva: World Health Organization; 2016.

[7] Fryar CD, Gu Q, Ogden CL, Flegal KM (2016). Anthropometric Reference Data for Children and Adults: United States, 2011–2014. Vital Health Stat 3 Anal Stud.

[8] Hales CM, Carroll MD, Fryar CD, Ogden CL (2020). Prevalence of obesity and severe obesity among adults: United States, 2017–2018. NCHS Data Brief.

[9] Anyanwu GO, Kolb AF, Bermano G. Antiobesity functional leads and targets for drug development. In: Egbuna C, Kumar S, Ifemeje JC, Ezzat SM, Kaliyaperumal S, editors. Phytochemicals as lead compounds for new drug discovery. Elsevier; 2020. p. 143–60.

[10] Busetto L, Sbraccia P, Vettor R (2022). Obesity management: at the forefront against disease stigma and therapeutic inertia. Eat Weight Disord.

[11] Daneschvar HL, Aronson MD, Smetana GW (2016). FDA-approved anti-obesity drugs in the United States. Am J Med.

[12] Müller TD, Blüher M, Tschöp MH, DiMarchi RD (2022). Anti-obesity drug discovery: advances and challenges. Nat Rev Drug Discov.

[13] Okoye NN, Ajaghaku DL, Okeke HN, Ilodigwe EE, Nworu CS, Okoye FB (2014). beta-Amyrin and alpha-amyrin acetate isolated from the stem bark of Alstonia boonei display profound anti-inflammatory activity. Pharm Biol.

[14] Akinloye O, Oshilaja R, Okelanfa O, Akinloye D, Idowu O (2013). Hypoglyceamic activity of Alstonia boonei stem bark extract in mice. Agric Biol J N Am.

[15] Onyeneke E, Anyanwu G. Anti-obesity potential of the ethanolic extract of Alstonia boonei stem bark on high carbohydrate diet induced obesity in male Wistar rats. NISEB J. 2018;14(1):46–50.

[16] Sarker SD, Latif Z, Gray AI. Natural product isolation: an overview. In: Sarker SD, Latif Z, Gray AI, editors. Natural Products Isolation, 2nd ed. Methods in Biotechnology, vol 20. Humana Press; 2006. p. 1–25.

[17] Anyanwu GO, Iqbal J, Khan SU, Zaib S, Rauf K, Onyeneke CE, Ojo OO, Nisar Ur R (2019). Antidiabetic activities of chloroform fraction of Anthocleista vogelii Planch root bark in rats with diet- and alloxan-induced obesity-diabetes. J Ethnopharmacol.

[18] Kim J, Jang DS, Kim H, Kim JS (2009). Anti-lipase and lipolytic activities of ursolic acid isolated from the roots of Actinidia arguta. Arch Pharm Res.

[19] Xiao Z, Storms R, Tsang A (2006). A quantitative starch-iodine method for measuring alpha-amylase and glucoamylase activities. Anal Biochem.

[20] Johnson MH, de Mejia EG, Fan J, Lila MA, Yousef GG (2013). Anthocyanins and proanthocyanidins from blueberry-blackberry fermented beverages inhibit markers of inflammation in macrophages and carbohydrate-utilizing enzymes in vitro. Mol Nutr Food Res.

[21] Yang Z, Tu Y, Xia H, Jie G, Chen X, He P (2007). Suppression of free-radicals and protection against H2O2-induced oxidative damage in HPF-1 cell by oxidized phenolic compounds present in black tea. Food chem.

[22] Roh C, Jung U (2012). Screening of crude plant extracts with anti-obesity activity. Int J Mol Sci.

[23] Morris GM, Huey R, Lindstrom W, Sanner MF, Belew RK, Goodsell DS, Olson AJ (2009). AutoDock4 and AutoDockTools4: Automated docking with selective receptor flexibility. J Comput Chem.

[24] Yamamoto K, Miyake H, Kusunoki M, Osaki S (2010). Crystal structures of isomaltase from saccharomyces cerevisiae and in complex with its competitive inhibitor maltose. Febs j.

[25] Heo L, Park H, Seok C (2013). GalaxyRefine: Protein structure refinement driven by side-chain repacking. Nucleic Acids Res.

[26] Wiederstein M, Sippl MJ. ProSA-web: interactive web service for the recognition of errors in three-dimensional structures of proteins. Nucleic Acids Res. 2007;35(suppl_2):W407–W410. 10.1093/nar/gkm290 PMC1933241 17517781

[27] Colovos C, Yeates TO (1993). Verification of protein structures: patterns of nonbonded atomic interactions. Protein Sci.

[28] Vaguine AA, Richelle J, Wodak SJ (1999). SFCHECK: a unified set of procedures for evaluating the quality of macromolecular structure-factor data and their agreement with the atomic model. Acta Crystallogr D Biol Crystallogr.

[29] O’Boyle N, Banck M, James CA, Morley C, Vandermeersch T (2011). Hutchison GR Open Babel: An open chemical toolbox. J Cheminf.

[30] Ogunyemi OM, Gyebi GA, Ibrahim IM, Esan AM, Olaiya CO, Soliman MM, Batiha GE (2023). Identification of promising multi-targeting inhibitors of obesity from Vernonia amygdalina through computational analysis. Mol Divers.

[31] Ogunyemi OM, Gyebi GA, Ibrahim IM, Olaiya CO, Ocheje JO, Fabusiwa MM, Adebayo JO (2021). Dietary stigmastane-type saponins as promising dual-target directed inhibitors of SARS-CoV-2 proteases: a structure-based screening. RSC adv.

[32] Trott O, Olson AJ (2010). AutoDock Vina: improving the speed and accuracy of docking with a new scoring function, efficient optimization, and multithreading. J Comput Chem.

[33] Ahmed B, Ali Ashfaq U, Usman Mirza M (2018). Medicinal plant phytochemicals and their inhibitory activities against pancreatic lipase: molecular docking combined with molecular dynamics simulation approach. Nat Prod Res.

[34] Kucio C, Jonderko K, Piskorska D (1991). Does yohimbine act as a slimming drug?. Isr J Med Sci.

[35] Jitomir J, Nassar E, Culbertson J, Moreillon J, Buford T, Hudson G, Cooke M, Kreider R, Willoughby DS (2008). The acute effects of the thermogenic supplement meltdown on energy expenditure, fat oxidation, and hemodynamic responses in young, healthy males. J Int Soc Sports Nutr.

[36] Anyanwu GO, Onyeneke EC, Okoli BJ, Johannes MS, Sabi RU, Iqbal J, Ejaz SA, Zaib S, Rauf K, Nisar RU (2019). Pharmacological activities of a novel phthalic acid ester and iridoid glycoside isolated from the root bark of Anthocleista vogelii Planch. Trop Biomed.

[37] Aboelnaga SMH (2021). Evaluation of the Antihyperlipidemic and antioxidant effects of Catharanthus roseus extracted from Vinca minor in diabetic rats. J Pharm Res Int.

[38] Liu TT, Liu XT, Chen QX, Shi Y (2020). Lipase inhibitors for obesity: a review. Biomed Pharmacother.

[39] Aabideen ZU, Mumtaz MW, Akhtar MT, Raza MA, Mukhtar H, Irfan A, Raza SA, Touqeer T, Nadeem M, Saari N (2021). Cassia fistula Leaves; UHPLC-QTOF-MS/MS Based Metabolite Profiling and Molecular Docking Insights to Explore Bioactives Role Towards Inhibition of Pancreatic Lipase. Plants (Basel).

[40] Aabideen ZU, Mumtaz MW, Akhtar MT, Raza MA, Mukhtar H, Irfan A, Raza SA, Nadeem M, Ling YS (2022). Anti-obesity effect and UHPLC-QTOF-MS/MS based metabolite profiling of Solanum nigrum leaf extract. Asian Pac J Trop Biomed.

[41] Bajes HR, Almasri I, Bustanji Y (2020). Plant products and their inhibitory activity against pancreatic lipase. Rev bras Farmacogn.

[42] Shi Z, Zhu Y, Teng C, Yao Y, Ren G, Richel A (2020). Anti-obesity effects of α-amylase inhibitor enriched-extract from white common beans (Phaseolus vulgaris L.) associated with the modulation of gut microbiota composition in high-fat diet-induced obese rats. Food Funct.

[43] Etesami B, Ghaseminezhad S, Nowrouzi A, Rashidipour M, Yazdanparast R (2020). Investigation of 3T3-L1 cell differentiation to adipocyte, affected by aqueous seed extract of phoenix dactylifera L. Rep Biochem Mol Biol.

[44] Li Y, Rong Y, Bao L, Nie B, Ren G, Zheng C, Amin R, Arnold RD, Jeganathan RB, Huggins KW (2017). Suppression of adipocyte differentiation and lipid accumulation by stearidonic acid (SDA) in 3T3-L1 cells. Lipids Health Dis.

[45] Nielsen TS, Jessen N, Jørgensen JO, Møller N, Lund S (2014). Dissecting adipose tissue lipolysis: molecular regulation and implications for metabolic disease. J Mol Endocrinol.

[46] Faraone I, Russo D, Genovese S, Milella L, Monné M, Epifano F, Fiorito S (2021). Screening of in vitro and in silico α-amylase, α-glucosidase, and lipase inhibitory activity of oxyprenylated natural compounds and semisynthetic derivatives. Phytochem.

[47] Rydberg EH, Li C, Maurus R, Overall CM, Brayer GD, Withers SG (2002). Mechanistic analyses of catalysis in human pancreatic alpha-amylase: detailed kinetic and structural studies of mutants of three conserved carboxylic acids. Biochem.

[48] Williams LK, Li C, Withers SG, Brayer GD (2012). Order and disorder: differential structural impacts of myricetin and ethyl caffeate on human amylase, an antidiabetic target. J Med Chem.

[49] Ogunyemi OM, Gyebi GA, Saheed A, Paul J, Nwaneri-Chidozie V, Olorundare O, Adebayo J, Koketsu M, Aljarba N, Alkahtani S (2022). Inhibition mechanism of alpha-amylase, a diabetes target, by a steroidal pregnane and pregnane glycosides derived from Gongronema latifolium Benth. Front Mol Biosci.

[50] Taha M, Noreen T, Imran S, Nawaz F, Chigurupati S, Selvaraj M, Rahim F, Hadiani Ismail N, Kumar A, Mosaddik A (2019). Synthesis, α-amylase inhibition and molecular docking study of bisindolylmethane sulfonamide derivatives. Med Chem Res.

[51] Guan T, Qian Y, Tang X, Huang M, Huang L, Li Y, Sun H (2011). Maslinic acid, a natural inhibitor of glycogen phosphorylase, reduces cerebral ischemic injury in hyperglycemic rats by GLT-1 up-regulation. J Neurosci Res.

[52] Liu J, Sun H, Duan W, Mu D, Zhang L (2007). Maslinic acid reduces blood glucose in KK-Ay mice. Biol Pharm Bull.

[53] Moorthy NS, Ramos MJ, Fernandes PA (2012). Studies on α-glucosidase inhibitors development: magic molecules for the treatment of carbohydrate mediated diseases. Mini Rev Med Chem.

[54] Rouzbehan S, Moein S, Homaei A, Moein MR (2017). Kinetics of α-glucosidase inhibition by different fractions of three species of Labiatae extracts: a new diabetes treatment model. Pharm Biol.

[55] Winkler FK, D'Arcy A, Hunziker W (1990). Structure of human pancreatic lipase. Nature.

[56] Nguyen PTV, Huynh HA, Truong DV, Tran TD, Vo CT (2020). Exploring Aurone derivatives as potential human pancreatic lipase inhibitors through molecular docking and molecular dynamics simulations. Molecules.

[57] Lüthi-Peng Q, Märki HP, Hadváry P (1992). Identification of the active-site serine in human pancreatic lipase by chemical modification with tetrahydrolipstatin. FEBS Lett.

[58] Hamden K, Jaouadi B, Zaraî N, Rebai T, Carreau S, Elfeki A (2011). Inhibitory effects of estrogens on digestive enzymes, insulin deficiency, and pancreas toxicity in diabetic rats. J Physiol Biochem.

[59] Hermans MM, Kroos MA, van Beeumen J, Oostra BA, Reuser AJ (1991). Human lysosomal alpha-glucosidase. Characterization of the catalytic site. J Biol Chem.

[60] Rahim F, Zaman K, Taha M, Ullah H, Ghufran M, Wadood A, Rehman W, Uddin N, Shah SAA, Sajid M (2020). Synthesis, in vitro alpha-glucosidase inhibitory potential of benzimidazole bearing bis-Schiff bases and their molecular docking study. Bioorg Chem.

