# Correction: Phytochemical analysis, in vitro and in silico effects from Alstonia boonei De Wild stem bark on selected digestive enzymes and adipogenesis in 3T3‑L1 preadipocytes

**DOI:** 10.1186/s12906-023-04236-w

**Published:** 2023-10-28

**Authors:** Gabriel O. Anyanwu, Uju D. Ejike, Gideon A. Gyebi, Khalid Rauf, Nisar-Ur-Rehman, Jamshed Iqbal, Sumera Zaib, Usunomena Usunobun, Eusebius C. Onyeneke, Badriyah S. Alotaibi, Gaber El-Saber Batiha

**Affiliations:** 1 Department of Biochemistry, Bingham University, Karu, Nasarawa State Nigeria; 2 Department of Pharmacy, COMSATS University Islamabad, Abbottabad Campus, Pakistan; 3 Centre for Advanced Drug Research, COMSATS University Islamabad, Abbottabad Campus, Pakistan; 4 Department of Basic and Applied Chemistry, Faculty of Science and Technology, University of Central Punjab, Lahore, 54590 Pakistan; 5 Department of Biochemistry, Faculty of Basic Medical Sciences, Edo University Uzairue, Auchi, Edo State Nigeria; 6 Department of Biochemistry, University of Benin, Benin City, Edo State Nigeria; 7 Department of Pharmaceutical Sciences, College of Pharmacy, Princess Nourah Bint Abdulrahman University, Riyadh, 11671 Saudi Arabia; 8 Department of Pharmacology and Therapeutics, Faculty of Veterinary Medicine, Damanhour University, Damanhour, 22511 AlBeheira Egypt

**Correction: BMC Complement Med Ther 23, 370 (2023)**

10.1186/s12906-023-04202-6

Following publication of the original article [1], the authors reported an error in Acknowledgment and Funding sections. The number 11671 in both sections should be removed.

The original article has been corrected.
