# Supplementary material for: Phytochemical analysis, in vitro and in silico effects from Alstonia boonei De Wild stem bark on selected digestive enzymes and adipogenesis in 3T3-L1 preadipocytes
Source: BMC Complement Med Ther. 2023 Oct 20;23:370. doi: 10.1186/s12906-023-04202-6 (PMC10588189; doi:10.1186/s12906-023-04202-6)
Supplement: Supplementary file 1 — Additional file 1: Figure SM1. Total Compound Chromatogram of crude alkaloid fraction of A. boonei via LCMS/MS analysis in negative polarity. The details of the numbers on each peak corresponding to the identified compounds from A. boonei are presented in Table SM1b. Figure SM2a. Saccharomyces cerevisiae α-glucosidase aligned with the template protein. Figure SM2b. Ramachandran phi/psi torsion angles plot of modelled Saccharomyces cerevisiae α-glucosidase. Figure SM2c. Errat plot of modeled Saccharomyces cerevisiae α-glucosidase. [file 12906_2023_4202_MOESM1_ESM.docx]

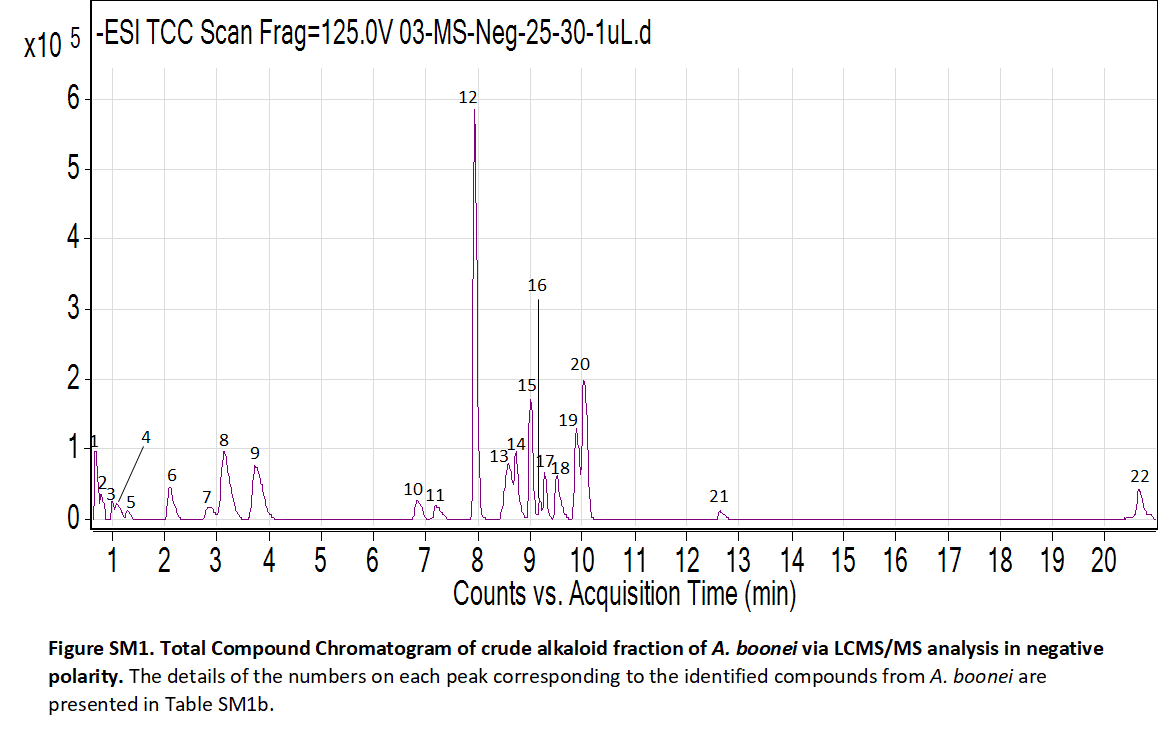


**Figure SM1. Total Compound Chromatogram of crude alkaloid fraction of *A. boonei* via LCMS/MS analysis in negative polarity.** The details of the numbers on each peak corresponding to the identified compounds from *A. boonei* are presented in Table SM1b.


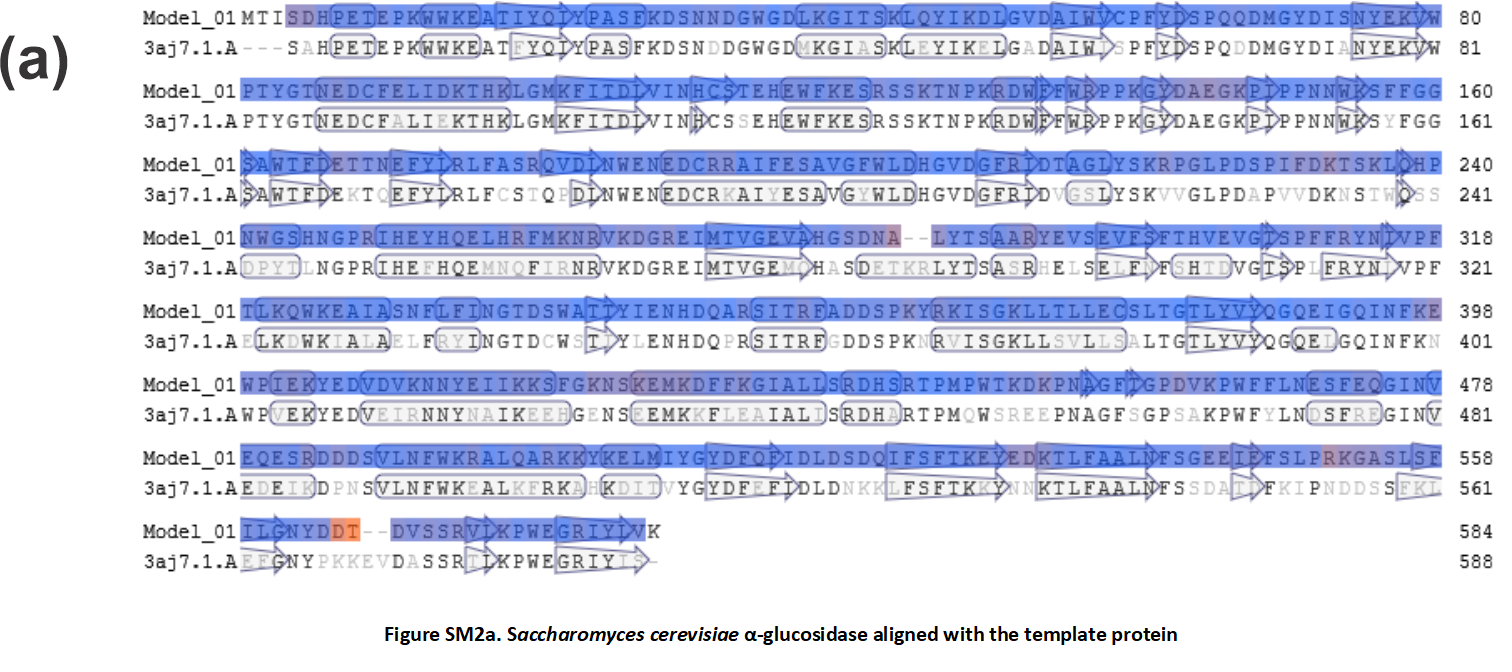


#### Figure SM2a *Saccharomyces cerevisiae* α-glucosidase aligned with the template protein. Amino acid sequence of modeled *Saccharomyces cerevisiae* α-glucosidase aligned with the sequence of isomaltase from *Saccharomyces cerevisiae* enzyme (PDB ID: 3A4A) used as template for the modeling. The modeled enzymes share 72.24% identity with the saccharomyces cerevisiae isomaltase.


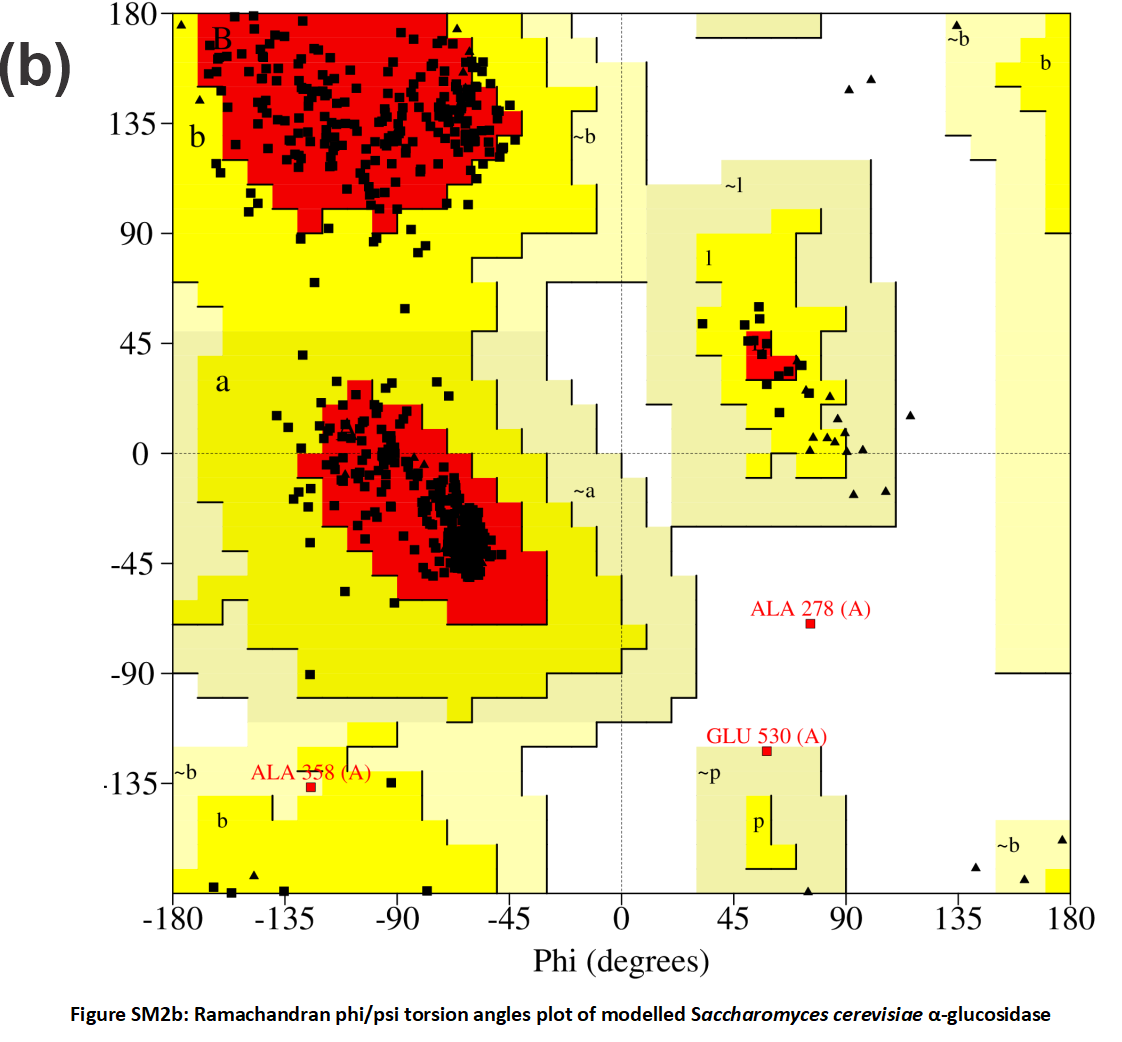


**Figure SM2b: Ramachandran phi/psi torsion angles plot of modelled S*accharomyces cerevisiae* α-glucosidase***.* 99.3% of amino acid residues were in favoured region with additional 11.1 % in allowed region, while 0.2% were in disallowed region.


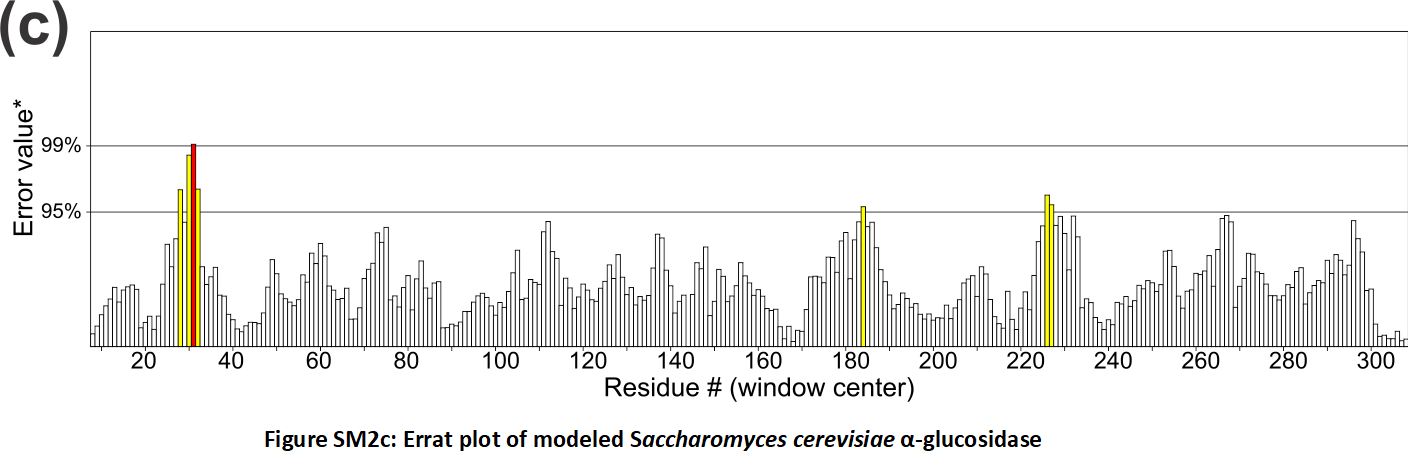


**Figure SM2c: Errat plot of modeled *Saccharomyces cerevisiae α-glucosidase.*** From the ERRAT plot analysis, the overall quality factor (OQF) of the model was 96.80% above the generally accepted score of >50%.
