# Supplementary material for: Phytochemical analysis, in vitro and in silico effects from Alstonia boonei De Wild stem bark on selected digestive enzymes and adipogenesis in 3T3-L1 preadipocytes
Source: BMC Complement Med Ther. 2023 Oct 20;23:370. doi: 10.1186/s12906-023-04202-6 (PMC10588189; doi:10.1186/s12906-023-04202-6)
Supplement: Supplementary file 2 — Additional file 2: Table SM1a. Chromatographic analysis of crude alkaloid fraction of A. boonei via positive polarity. Table SM1b. Compounds result of the LCMS/MS analysis (negative mode) of crude alkaloid fraction of A. boonei. Table SM1c. Chromatographic analysis of crude alkaloid fraction of A. boonei via negative polarity. Table SM2. Chromatographic analysis of crude saponin fraction of A. boonei via negative polarity. Table SM3. Binding energies of LCMS/MS identified compounds from the fractions of A. boonei docked in the active sites of human α-amylase, lipase and α-glucosidase. Table SM4. Top two ranked compounds from the molecular docking of the LCMS/MS identified compounds from the crude. [file 12906_2023_4202_MOESM2_ESM.zip › Table SM1b.docx]

**Table SM1b. Compounds result of the LCMS/MS analysis (negative mode) of crude alkaloid fraction of *A. boonei***

| Peak | RT | Mass | Name | Formula | Sub-class | Class |
| --- | --- | --- | --- | --- | --- | --- |
| 1 | 0.71 | 136.04 | Hypoxanthine | C_5_ H_4_ N_4_ O | Purines and purine derivatives | Xanthine |
| 2 | 0.72 | 132.04 | Pteridine | C_6_ H_4_ N_4_ | Azaarene | Organic heterocyclic compound |
| 3 | 1.00 | 129.04 | N-Acryloylglycine | C_5_ H_7_ N O_3_ | Amino acids, peptides, and analogues | Carboxylic acids and derivatives |
| 4 | 1.16 | 148.04 | 2-Propenyl propyl disulfide | C_6_ H_12_ S_2_ | Allyl sulfur compounds | Sulfides |
| 5 | 1.29 | 148.04 | (R)-2-Methylmalate | C_5_ H_8_ O_5_ | Citramalic acid | Carboxylic acid |
| 6 | 2.10 | 188.10 | Nonic Acid | C_9_ H_16_ O_4_ | Fatty acids and conjugates | Fatty Acyls |
| 7 | 2.87 | 214.08 | Unknown | C_10_ H_14_ O_5_ |  |  |
| 8 | 3.15 | 186.09 | cis-2-Carboxycyclohexyl-acetic acid | C_9_ H_14_ O_4_ | - | Carboxylic acid |
| 9 | 3.77 | 186.09 | 5-(2-Methylpropyl)tetrahydro-2-oxo-3-furancarboxylic acid | C_9_ H_14_ O_4_ | Gamma butyrolactones | Lactones |
| 10 | 6.85 | 246.11 | Unknown | C_11_ H_18_ O_6_ |  |  |
| 11 | 7.29 | 176.10 | Metaldehyde | C_8_ H_16_ O_4_ | - | Aldehydes |
| 12 | 7.93 | 404.13 | Gardenoside | C_17_ H_24_ O_11_ | Iridoids | Monoterpenes |
| 13 | 8.54 | 430.21 | Cinegalline | C_23_ H_30_ N_2_ O_6_ | Quinolizidine alkaloid | Alkaloid |
| 14 | 8.59 | 400.20 | Reserpic acid | C_22_ H_28_ N_2_ O_5_ | Yohimbine | Alkaloids |
| 15 | 9.01 | 368.17 | Horhammericine | C_21_ H_24_ N_2_ O_4_ | Indole alkaloids | Alkaloids |
| 16 | 9.18 | 430.21 | Cinegalline | C_23_ H_30_ N_2_ O_6_ | Quinolizidine alkaloid | Alkaloid |
| 17 | 9.28 | 446.14 | Lucuminic acid | C_19_ H_26_ O_12_ | Glycosides | Carbohydrates and carbohydrate conjugates |
| 18 | 9.50 | 382.19 |  | C_17_ H_27_ Cl N_6_ O_2_ |  |  |
| 19 | 9.89 | 184.07 | (±)-threo-1-(4-Hydroxyphenyl)-1,2,3-propanetriol | C_9_ H_12_ O_4_ | 1-hydroxy-2-unsubstituted benzenoids | Phenols |
| 20 | 10.03 | 142.10 | Unknown | C_8_ H_14_ O_2_ |  |  |
| 21 | 12.64 | 222.13 | Hydroxyibuprofen | C_13_ H_18_ O_3_ | Phenylpropionates | Carboxylic acids |
