# Supplementary material for: Phytochemical analysis, in vitro and in silico effects from Alstonia boonei De Wild stem bark on selected digestive enzymes and adipogenesis in 3T3-L1 preadipocytes
Source: BMC Complement Med Ther. 2023 Oct 20;23:370. doi: 10.1186/s12906-023-04202-6 (PMC10588189; doi:10.1186/s12906-023-04202-6)
Supplement: Supplementary file 2 — Additional file 2: Table SM1a. Chromatographic analysis of crude alkaloid fraction of A. boonei via positive polarity. Table SM1b. Compounds result of the LCMS/MS analysis (negative mode) of crude alkaloid fraction of A. boonei. Table SM1c. Chromatographic analysis of crude alkaloid fraction of A. boonei via negative polarity. Table SM2. Chromatographic analysis of crude saponin fraction of A. boonei via negative polarity. Table SM3. Binding energies of LCMS/MS identified compounds from the fractions of A. boonei docked in the active sites of human α-amylase, lipase and α-glucosidase. Table SM4. Top two ranked compounds from the molecular docking of the LCMS/MS identified compounds from the crude. [file 12906_2023_4202_MOESM2_ESM.zip › Table SM4.docx]

Table SM4: Top two ranked compounds from the molecular docking of the LCMS/MS identified compounds from the crude alkaloid and saponin fraction of *A. boonei* against α-amylase, lipase and α-glucosidase

| S/No | Compound |  |
| --- | --- | --- |
|  | Orlistat |  |
|  | Acarbose |  |
| 1 | 3α-O-trans-Feruloyl-2 α-  hydroxy-12-ursen-28-oic acid |  |
| 2 | Estradiol-17-phenylpropionate |  |
| 3 | 10-Hydroxyyohimbine |  |
